# Plasma degradome affected by variable storage of human blood

**DOI:** 10.1186/s12014-016-9126-9

**Published:** 2016-09-26

**Authors:** Maria Kaisar, Leon F. A. van Dullemen, Marie-Laëtitia Thézénas, M. Zeeshan Akhtar, Honglei Huang, Sandrine Rendel, Philip D. Charles, Roman Fischer, Rutger J. Ploeg, Benedikt M. Kessler

**Affiliations:** 1 Nuffield Department of Surgical Sciences, University of Oxford, Oxford, OX3 7LJ UK; 2 NHS Blood and Transplant, Watford, WD24 4QN UK; 3 Surgical Research Laboratory, University Medical Center, University of Groningen, Groningen, 9713 GZ The Netherlands; 4 Target Discovery Institute, Nuffield Department of Medicine, University of Oxford, Oxford, OX3 7FZ UK

**Keywords:** Pre analytical variability, Ambient temperature, Plasma proteome, PROTOMAP, Mass spectrometry, Biobank, QUOD

## Abstract

**Background:**

The successful application of—omics technologies in the discovery of novel biomarkers and targets of therapeutic interventions is facilitated by large collections of well curated clinical samples stored in bio banks. Mining the plasma proteome holds promise to improve our understanding of disease mechanisms and may represent a source of biomarkers. However, a major confounding factor for defining disease-specific proteomic signatures in plasma is the variation in handling and processing of clinical samples leading to protein degradation. To address this, we defined a plasma proteolytic signature (degradome) reflecting pre-analytical variability in blood samples that remained at ambient temperature for different time periods after collection and prior to processing.

**Methods:**

We obtained EDTA blood samples from five healthy volunteers (n = 5), and blood tubes remained at ambient temperature for 30 min, 8, 24 and 48 h prior to centrifugation and isolation of plasma. Naturally occurred peptides derived from plasma samples were compared by label-free quantitative LC–MS/MS. To profile protein degradation, we analysed pooled plasma samples at T = 30 min and 48 h using PROTOMAP analysis. The proteolytic pattern of selected protein candidates was further validated by immunoblotting.

**Results:**

A total of 820 plasma proteins were surveyed by PROTOMAP, and for 4 % of these, marked degradation was observed. We show distinct proteolysis patterns for talin-1, coagulation factor XI, complement protein C1r, C3, C4 and thrombospondin, and several proteins including S100A8, A9, annexin A1, profiling-1 and platelet glycoprotein V are enriched after 48 h blood storage at ambient temperature. In particular, thrombospondin protein levels increased after 8 h and proteolytic fragments appeared after 24 h storage time.

**Conclusions:**

The overall impact of blood storage at ambient temperature for variable times on the plasma proteome and degradome is relatively minor, but in some cases can cause a potential bias in identifying and assigning relevant proteomic markers. The observed effects on the plasma proteome and degradome are predominantly triggered by limited leucocyte and platelet cell activation due to blood handling and storage. The baseline plasma degradome signature presented here can help filtering candidate protein markers relevant for clinical biomarker studies.

**Electronic supplementary material:**

The online version of this article (doi:10.1186/s12014-016-9126-9) contains supplementary material, which is available to authorized users.

## Background

The successful application of—omics technologies in medical research for discovering disease-specific molecular processes is facilitated by access to large collections of well curated clinical samples available for analysis. Biobanks as a source of biological samples with associated clinical and demographic data are essential for the study of disease mechanisms and for the discovery of novel biomarkers and targets of therapeutic interventions [1]. For many discovery studies, longitudinal blood, urine samples and organ biopsies are collected, processed and stored according to detailed standard operating protocols within hospitals. Often, guidelines suggest that blood samples should be transferred on dry ice to prevent protein degradation [2, 3]. We recently established a large UK biobank, within the framework of NHS Blood and Transplant, collecting samples from 60 national wide sites in organ donation and transplantation (QUOD) [4]. The purpose of UK QUOD biobank is to provide a comprehensive collection of clinical samples, obtained during deceased donor organ management, for research on organ donation and transplantation. In developing the protocols for QUOD, we realised that there is a lack of a consensus in the collection, processing and storage protocols for blood, urine, other body fluids or tissue biopsies not only relevant for QUOD specifically, but also for other biobanks in general. For blood in particular, variables due to sample handling have been described [5] which may differ in the context of measuring specific clinical parameters such as vitamin E (alpha-tocopherol) [6] glucose [7], but also for immunoassays [8], blood based amyloid-beta assays [9], C-reactive protein CRP [10] or sCD40L [11]. Recently, proteomic technologies have been employed for the discovery of biomarkers and novel targets of interventions in diverse fields of medicine. In such studies, there are many variables that can influence the outcome of mass spectrometry based serum/plasma proteomics [12, 13]. Eliminating sample variability is particularly important to reduce false-positive discovery of potential biomarkers [14, 15]. Recommendations for sample processing vary from immediate storage of blood on ice to storage and processing at ambient temperature. Lower temperatures reduce partial degradation of plasma proteins but lead to an increase in proteins related to platelet activation and coagulation [16, 17]. We reasoned that cooling samples may lead to undesirable artefacts and more variability, thereby introducing a bias to the observed results. We are therefore favouring blood sample handling at ambient temperature for whole blood collection and previously showed that the plasma proteome is remarkable stable in AT following blood collection [18]. Protein degradation (degradome) or the peptidome (naturally occurring peptide fragments) has been assessed in plasma, in particular in the context of coagulation [19], and proteolytic fragments derived from fibrinogen alpha chain, apolipoprotein A-IV, A-I, complement C3 and alpha-1 antitrypsin can be readily detected, and they were reported to correlate with tumorigenesis but potentially confounded with clotting [15]. As keeping blood at ambient temperature does minimise platelet and complement activation and since these conditions are more applicable in a clinical setting, we set out to systematically profile the plasma degradome at variable blood storage times.

## Methods

### Sample collection and processing

**Fig. 1:**
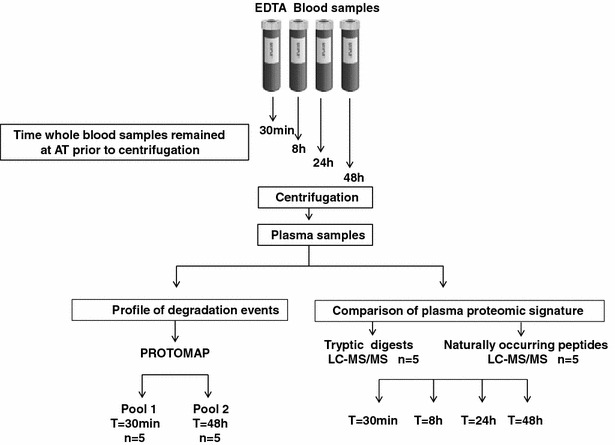
*Experimental workflow.* Four EDTA blood tubes were collected from five healthy volunteers (n = 5) and remained at ambient temperature for T = 30 min, 8, 24 or 48 h before centrifugation, processing and analysis by liquid chromatography tandem mass spectrometry (LC–MS/MS). Comparison of plasma proteomic signatures of individual samples by LC–MS/MS of tryptic (reflecting proteins) and naturally occurred peptides (peptidome) in blood performed at the indicated time points. Profiling of protein degradation (degradome) in plasma from pooled blood samples (n = 5) collected at 30 min and 48 h was performed and subsequently analysed using PROTOMAP

We collected 40 ml blood from five healthy individuals in EDTA gel vacutainer plasma gel separator tubes (BD, Vacutainer^®^ PPTTM Plastic tube with BD Pearlescent White HemogardTM Closure). Blood samples were collected from healthy volunteers within Oxford University according to the research consent policy [20]. Blood was collected by peripheral venepuncture using a 20-gauge needle and was mixed with EDTA by gently inverting the EDTA tubes, followed by storage at ambient temperature ~22 °C (*AT*) for 30 min, 8, 24, or 48 h (Fig. 1). For the purpose of this study, ambient temperature (*AT*) was defined as 22 ± 2 °C, and the protocols for preparation of all the samples were the same for each participant. Subsequently, plasma was prepared by centrifugation at 1500×*g* for 15 min at 22 °C. Plasma supernatant was aliquoted and stored at −80 °C until further analysis. No haemolysis was observed in any of the blood samples before or after blood centrifugation or during the period of 48 h at ambient temperature. Plasma samples were immunodepleted of highly abundant proteins prior to further processing as described below.

### Plasma depletion of highly abundant proteins

Antibody affinity-based depletion of high abundance proteins present in human plasma was conducted using an Agilent Human top 14 Multiple Affinity Removal System (MARS) coupled to an Ultimate 3000 HPLC system (Thermo Scientific) following manufacturer’s instructions. Briefly, 80 µl plasma aliquots were centrifuged at 10,000×*g* for 10 min, diluted four times in Buffer A (Agilent Technologies, UK) and separated on the MARS column according to the manufacturer’s instructions. Protein depletion followed a sequence of isocratic elution steps: 100 % buffer A for 20 min at 0.125 ml/min followed by 0.7 ml/min for 2.5 min. Flow-through fractions containing the depleted plasma were collected between 7.5 and 14.5 min of each sample run. Between runs, the column was washed with buffer B (Agilent Technologies, UK) until the UV_214nm_ trace was back to baseline. Each sample was injected four times to obtain sufficient quantity of protein for further analysis.

### Protein precipitation of individual plasma samples

Flow-through protein fractions of depleted plasma samples were precipitated with the addition of sodium deoxycholate to a final concentration of 125 µg/ml followed by 15 min incubation at 22 °C. Trichloroacetic acid was added to a final concentration of 6 %, followed by centrifugation at 12,000×*g* at 4 °C for 30 min. Following centrifugation, sample supernatants containing naturally occurring peptides were collected in new tubes for separate analyses. Protein precipitates were washed with ice-cold acetone, centrifuged at 12,000×*g* for further 10 min and pellets resuspended in 50 µl of 6 M urea in 100 mM Tris HCl (pH 7.8). Quantitation of each sample was performed by a BCA protein assay according to the manufacturer’s instructions (Thermo Scientific, BCA UK) and 80 Âµg of protein per sample was analysed (Fig. 1). Protein precipitates and naturally occurred peptides were further processed and subjected to label-free semi-quantitative liquid chromatography tandem mass spectrometry (LC–MS/MS) and PROTOMAP analysis as described below.

### Plasma proteome analysis

15 µg of precipitate protein material was used for each individual sample. Samples were reduced for 1 h by addition of 200 mM dithiothreitol (DTT) followed by alkylation with 200 mM iodoacetamide (IAA) for 30 min. Trypsin digestion was performed overnight at 37 °C with gentle mixing using a 1:50 (trypsin:protein) ratio. Samples were acidified with 1 % FA or TFA. Peptide digests were then desalted using Sep-Pak C18 cartridges (Waters) and dried by Speed Vac centrifugation. Pellets were resuspended in 30 µl of buffer A (98 % Milli-Q-H2O, 2 % acetonitrile, 0.1 % formic acid) and kept at −20 °C until analysis. Peptides were analysed in duplicates by nano ultra-high performance liquid chromatography tandem mass spectrometry (nUHPLC–MS/MS) using a Dionex Ultimate 3000 UHPLC (C18 column with a 75 μm × 250 mm, 1.7 μm particle size, Thermo Scientific, Bremen, Germany) coupled to a Q Exactive tandem mass spectrometer (Thermo Scientific, Bremen, Germany) as described previously [21].

### Proteomic analysis of naturally occurred peptides in plasma

Naturally occurred plasma peptides present in supernatant fractions after protein precipitation were purified using Sep-Pak C18 cartridges according to the manufacturer’s instructions. In brief, solid phase cartridges were equilibrated in 98 % Acetonitrile (ACN), 0.1 % Formic Acid (FA) and 2 % ACN, 0.1 % FA. Samples were then loaded onto the cartridge followed by washing with 2 % Acetonitrile (ACN), 0.1 % Formic Acid (FA) solution and subsequent peptide elution using 50 % ACN, 0.1 % FA. Peptide fractions were dried by vacuum centrifugation overnight. Pellets were resuspended in 20 µl of buffer A (98 % Milli-Q-H_2_O, 2 % acetonitrile, 0.1 % formic acid) and analysed by nUHPLC–MS/MS in duplicates as described above.

### Data analysis for plasma proteome

Raw MS data were processed using MSConvert v3.0.7529 (ProteoWizard) and analysed using Progenesis QI for Proteomics (QIP) software v3.1.4003.30577 (Nonlinear Dynamics). MS/MS spectra were searched against the UniProt Homo Sapiens Reference proteome (retrieved 15/10/2014) using Mascot v2.5.1 (Matrix Science) allowing for a precursor mass tolerance of 10 ppm and a fragment ion tolerance of 0.5 Da, Carbamidomethylation on Cysteines as fixed, and Deamidation (Glutamine) and Oxidation (Methionine) as variable modifications with a false discovery rate (FDR) of 1 %. Mascot results were imported into Progenesis QIP. Only proteins that were defined with at least 2 unique peptides were included in the protein data set for further analysis. Statistical comparison of protein abundance changes observed between the four time points of whole blood centrifugation (T = 30 min and T = 8, 24, and 48 h) was performed using a one-way ANOVA F-test within the Progenesis QI software (calling significant changes at *p* ≤ 0.05).

### Data analysis for naturally occurred peptides in plasma

Sequence interpretation of MS/MS spectra of naturally occurred peptides was performed by the interrogation of UniProt Homo Sapiens database using PEAKS Online v7.5 (Bioinformatics Solutions Inc.) with an FDR of 1 % and imported into the Progenesis QI software for quantitation. Average normalised abundance values were obtained via quantile normalisation using the Progenesis IQ software. For proteins, precursor ion intensities of unique tryptic peptides matched to the protein sequence were used. Only proteins that were defined with at least two unique peptides were included in the protein data set for further analysis. For naturally occurring peptides, individual precursor ion counts were used for quantitation and average normalised abundances obtained as described above. Statistical comparison of protein abundance changes observed between the four time points (n = 5 each) of whole blood centrifugation (T = 30 min and T = 8, 24, and 48 h) was performed using a one-way ANOVA F-test within the Progenesis QI software (calling significant changes at *p* ≤ 0.05).

### PROTOMAP analysis

To profile protein degradation events occurring in whole blood exposed to *AT* for different times, we used a PROTOMAP approach as described previously [22]. For this analysis, we created two plasma pools obtained from 5 healthy individuals as described in Fig. 1. Pool 1 contained plasma samples prepared from whole blood by centrifugation after 30 min following blood collection and Pool 2 contained plasma samples prepared 48 h after collection. After depletion of the most abundant plasma components (see above), 60 µg of protein per pool was reduced in standard Laemmli buffer with DTT, divided into two aliquots and separated by SDS–PAGE (NOVEX Invitrogen 4–12 % gradient, Thermo Scientific, Bremen, Germany). The gel was stained with Coomassie blue and each pooled sample lane was cut into 22 horizontal slices, generating 44 samples overall (Fig. 1). Each sample was subjected to in-solution trypsin digestion as described previously [23]. In brief, gel pieces were de-stained in a solution of 1 ml 50 % methanol, 5 % acetic acid in Milli-Q-H_2_O solution until transparent, then dehydrated using 200 µl ACN for 5 min. Proteins in gel pieces were reduced by addition of 30 µl of 10 mM dithiothreitol (DTT) for 30 min followed by alkylation with 30 µl of 50 mM iodoacetamide (IAA) for 30 min. Gel pieces were dehydrated with 200 µl ACN, resuspended in 30 µl 100 mM ammonium bi-carbonate containing 20 ng/µl trypsin and incubated overnight at 37 °C with gentle mixing. Peptide digests were extracted from the gel matrix using 50 µl extraction buffer I (50 % ACN, 5 % FA) followed by 50 µl extraction buffer II (85 % ACN, 5 % FA), collected and dried by vacuum centrifugation. Pellets were resuspended in 30 µl of buffer A (98 % Milli-Q-H2O, 2 % acetonitrile, 0.1 % formic acid) and analysed by nUHPLC–MS/MS using a Thermo LTQ Q Exactive tandem mass spectrometer as described previously [24].

### Analysis of PROTOMAP derived mass spectrometry data

**Fig. 2:**
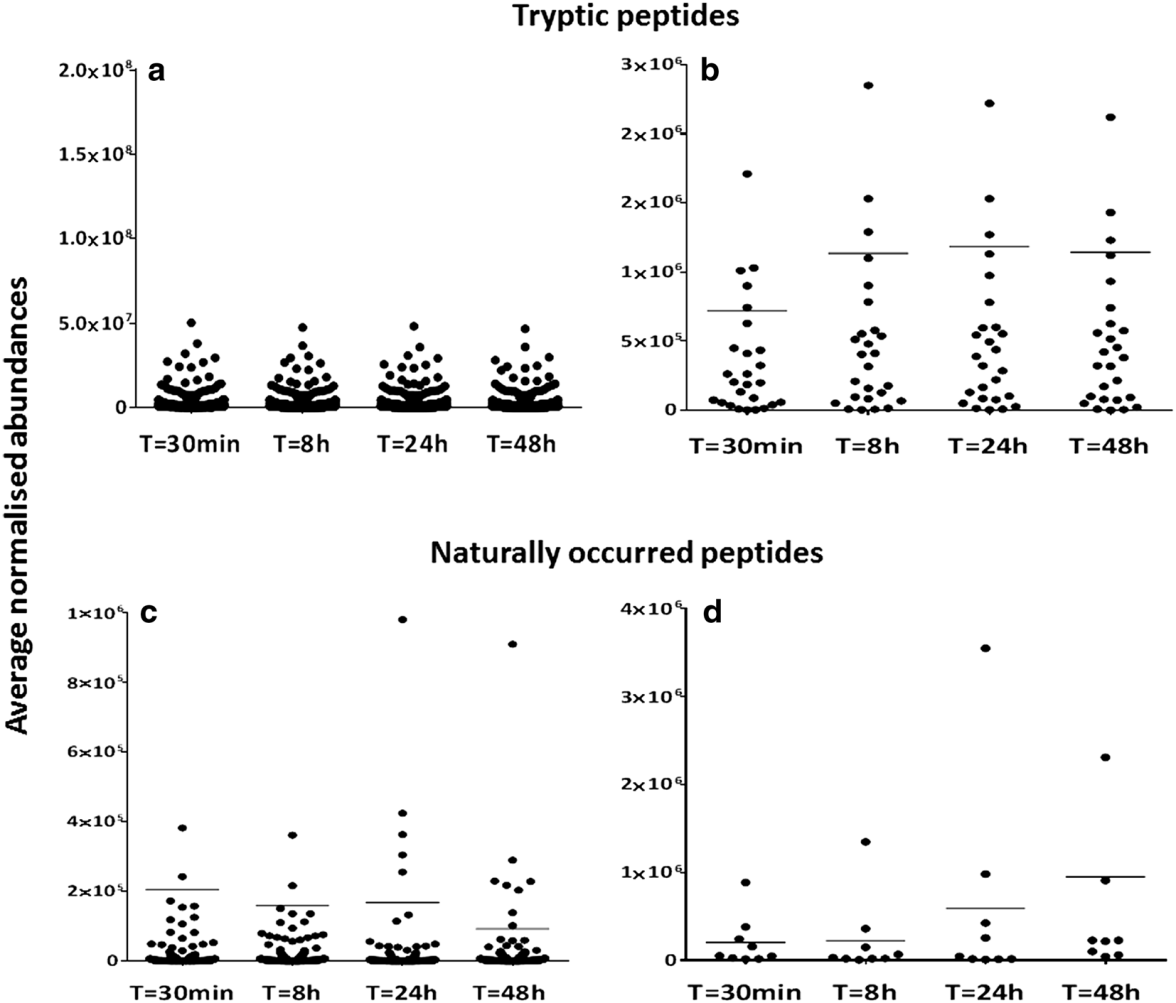
*Effect of blood storage on plasma proteome and peptidome signatures.* Plasma protein levels stay essentially constant upon blood stored at ambient temperature for different times. **a** Abundance levels of all proteins (represented by their corresponding tryptic peptides) identified at the indicated times (Additional file 1: Table S1). **b** Abundance levels of proteins showing > twofold change (ANOVA *p* < 0.05). **c** Abundance levels of 140 naturally occurred peptides (peptdidome) in plasma observed at the indicated times. **d** Abundance levels of naturally occurring peptides in plasma showing > twofold change (ANOVA *p* < 0.05)

The PROTOMAP integrates the protein migration patterns on SDS–PAGE electrophoresis with peptide sequence coverage and spectral counts acquired by LC–MS/MS analysis, and the results are visualised as peptographs (Fig. 2) [22]. In brief, raw data was converted to Mascot generic files using msconvert [25], searched with Mascot and further analysed as described by Niessen et al, [19]. To expound, MS/MS spectra data were searched using Mascot v2.5.1 against the UniProt *Homo sapiens* Reference proteome (retrieved 15/10/2014). The Mascot results were exported as DTASelect at an FDR threshold of 1 %, and analysed using the PROTOMAP perl scripts obtained from http://www.scripps.edu/cravatt/protomap/. Peptographs consist of two panels and combine information on 1-D gel migration of protein fragments, protein sequence coverage and spectral counts of protein fragments per gel band. The left panel shows the protein sequence coverage from N to C terminus in each band. Peptide sequences represented in red and blue were identified in the 30 min and 48 h pools, respectively, while peptide fragments represented in purple were common to both pooled samples in the same band. The right panel shows the relative quantitation using spectral counts for each protein between the two pools. The red bars represent the “parent proteins” which are the intact proteins that were identified in the plasma samples prepared after centrifugation at T = 30 min while blue bars represent the spectral counts of proteins in the samples centrifuged T = 48 h following collection. Protein degradation is defined by fragments with spectral counts detected in lower molecular weight bands as compared to the expected size of the “parent protein”.

### Western blot validation

Depleted plasma samples containing 20 µg of protein were denatured at 95 °C for 5 min in Laemmli buffer, loaded onto and separated by 4–12 % pre-cast SDS-PAGE gels (Bio-Rad, USA), followed by immunoblotting onto PVDF membranes (Merck Millipore, USA) using standard protocols. Membranes were incubated overnight at 4 °C with monoclonal rabbit anti-C4B (1 µg/ml, Abcam Ab168358) or goat polyclonal anti thrombospondin-1 (1.2 µg/ml, R&D systems AF3074). 1:5000 dilution of Dye-800-conjugated anti-goat or -rabbit IgG (Li-Cor, Nebraska, USA) were used as secondary antibodies for detection. Bands were detected using an Odyssey CLx system (Li-Cor Nebraska, USA).

## Results

### Plasma proteome and peptidome signatures of stored blood

To establish how the plasma proteome and degradation profile was affected by leaving whole blood at ambient temperature for a period of 30 min up to 48 h, we analysed plasma collected from 5 individuals whose blood was stored at ambient temperature for 30 min, 8, 24 and 48 h, respectively (Fig. 1). Interestingly, LC–MS/MS analysis revealed no obvious trend of change in the majority of protein levels with respect to time (at least 2 unique peptides and > 95 %confidence in protein identification) (Fig. 2a, b, Additional file 1: Table S1 for the list of all identified proteins). Next, we established how the plasma degradome profile was affected by storage via the analysis of naturally occurring peptides in plasma collected from five individuals whose blood was stored at ambient temperature for 30 min, 8, 24 and 48 h (Figs. 1, 2). Interestingly, LC–MS/MS analysis revealed only minor changes in peptides assigned to 140 plasma proteins (Fig. 2c). There was a significant increase in proteolytic peptides derived from nine proteins after 48 h. These included complement C4 and the cytoskeletal proteins vinculin, importin and filamin (Fig. 2d).

### PROTOMAP reveals minimal storage related degradation of plasma proteins

**Fig. 3:**
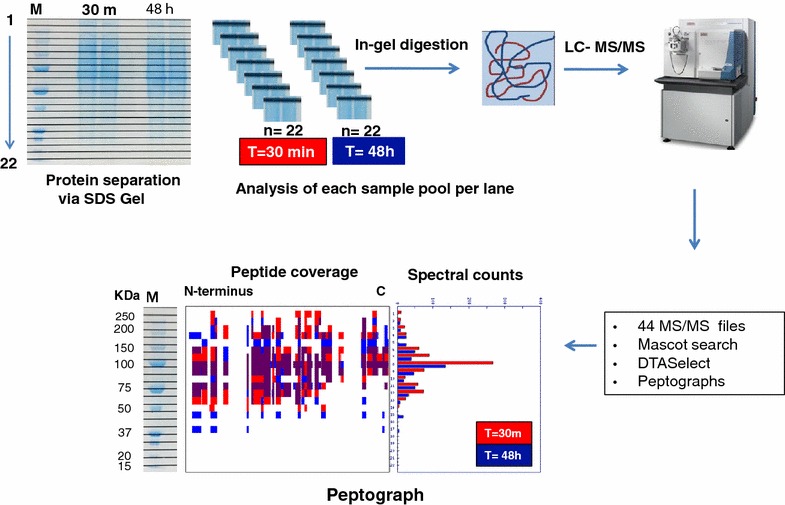
*PROTOMAP workflow.* The 30 min (Pool 1, n = 5) and 48 h samples (Pool 2, n = 5) were separated by 1-D SDS-PAGE and proteins visualised by Coomassie blue staining. The gel was subsequently divided into 22 bands per lane (representing one condition each). Each pool per band was cut to create n = 22 pieces per condition, proteins were subjected to in-solution trypsin digestion and analysed by LC-MS/MS. Raw MS data was analysed by PROTOMAP bioinformatics to generate peptographs [22]

**Fig. 4:**
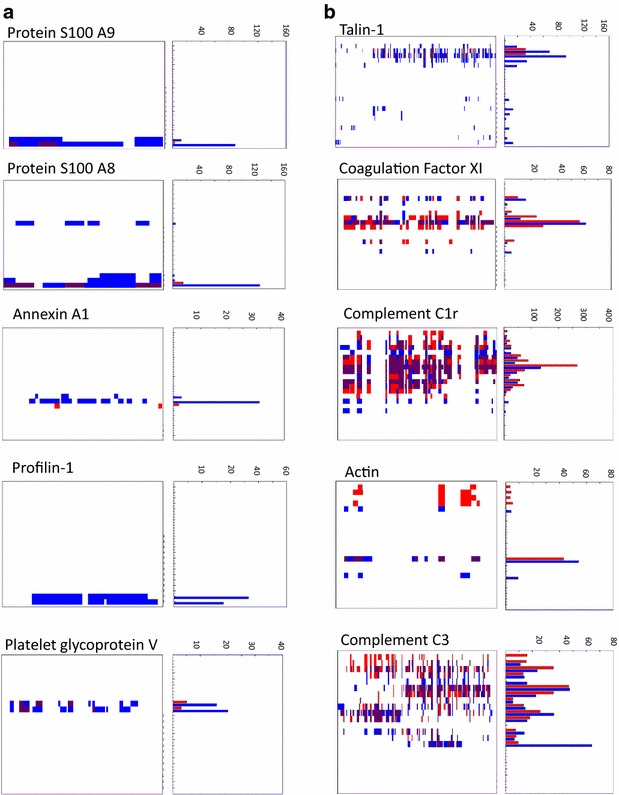
*PROTOMAP indicates plasma protein enrichment or degradation as a function of variable blood storage.* **a** Protein S100 A9, S100 A8, annexin A1, profiling-1 and platelet glycoprotein V levels are enriched after 48 h of blood storage (*blue bars*) as compared to 30 min (*red bars*). **b** Prolonged blood storage provokes partial degradation of talin-1, coagulation factor XI, complement C1r, C3 and actin as exemplified by their corresponding peptographs (*blue bars*—30 min; *red bars*—48 h blood storage at ambient temperature)

**Fig. 5:**
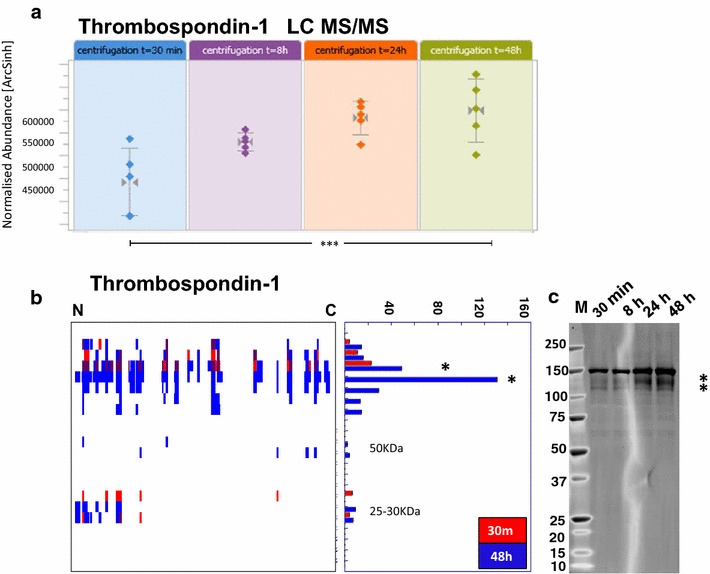
*Prolonged blood storage affects TSP*-*1 protein levels and degradation.* **a** Gradual increase of TSP-1 protein levels as indicated by label-free quantitative mass spectrometry analysis of tryptic peptides, indicating a 1.3-fold change after 48 h of blood storage (*p* < 0.001). **b** TSP-1 protein degradation patterns as observed by the PROTOMAP peptograph (*red bars*—30 min; *blue bars*—48 h) and confirmed by western blot analysis at the indicated times. The *stars* indicated in the PROTOMAP peptograph correspond to the bands in the 120–150 kDa region observed in the western blot, suggesting an increase in protein levels as well as partial degradation

**Fig. 6:**
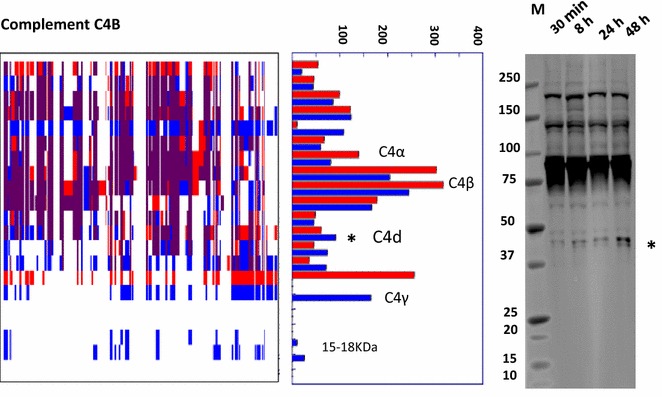
*Minimal proteolysis of complement C4B upon blood storage.* Blood sample pools centrifuged after 30 min and 48 h of storage at ambient temperature were analysed by PROTOMAP (*red bars*—30 min; *blue bars*—48 h) and anti-complement C4B western blotting. The 40 kDa fragment highlighted with a star (*) was increased as shown by the PROTOMAP peptograph, corresponding to a degradation intermediate also detected by western blotting. This fragment corresponds to the C4d component. Two more fragments of lower MW were increased in the PROTOMAP peptograph, corresponding to ~18 and ~30 kDa

To further map the proteolytic fragments of plasma proteins in whole blood stored at ambient temperature in greater detail, we applied a PROTOMAP approach. Pooled plasma samples (n = 5) derived from the blood storage conditions of 30 min and 48 h were compared and analysed (Fig. 3). The PROTOMAP method compares two conditions (control vs experimental), hence the requirement for pooling [22]. PROTOMAP analysis identified overall 820 proteins and notably only 52 proteins revealed changes between the T = 30 min and T = 48 h plasma pools. Shortlisted proteins (visualised as peptographs) showed patterns of degradation or enrichment, and their selection was based on either (i) increased spectral counts of fragments that had migrated at a lower molecular weight region than the intact protein or (ii) being enriched or uniquely identified at 48 h (Figs. 4, S1). From the 52 proteins, 20 were enriched in plasma, 22 showed breakdown products indicating likely proteolysis and for 10 proteins, we observed a combination of enrichment and degradation. Examples of peptographs are shown in Figs. 4, Additional file 1: S1 and S2. Peptographs of S100A8, S100A9, annexin A1, platelet glycoprotein 4 and profilin-1 demonstrate enrichment of these proteins at 48 h as were identified in the expected molecular weight at 10, 13, 38, 15 and 75 kDa, respectively, for each protein (Fig. 4a). Talin-1 showed increased fragmentation of the intact protein (>250 kDa) over time with breakdown intermediates observed at 140 kDa, 30–40 kDa and 15–22 kDa at 48 h. Coagulation factor XI (CFXI) and complement C3 show no protein enrichment but the appearance of degradation fragments at ~8 kDa for CXI and C3 (Fig. 4b). We also observed degradation profiles for fibrinogen alpha chain, serpin A3/A4, EMC1, ceruloplasmin, plasminogen-like protein A, PON1, ITIH1, fibronectin, apolipoprotein B-100 (Additional file 1: Figure S1) and complement C2/C5 (Additional file 1: Figure S2). In all these cases, the appearance of lower molecular weight protein fragments increases after blood storage for 48 h. Protein degradation profiled by PROTOMAP was validated by western blotting for thrombospondin 1 (TSP-1) and complement C4B. TSP-1 increased gradually to a maximum fold of 1.4 in the 48 h condition as compared to 30 min, consistent with LC–MS/MS analysis (Fig. 5a). PROTOMAP showed an increase of the intact protein (~150 kDa) and the appearance of protein fragments within the 80–150 kDa range and an N-terminal proteolytic fragment of ~25 kDa (Fig. 5b). Western blot validation of plasma TSP-1 for all four conditions indicated a gradual increase in TSP-1 protein levels correlating with the time of blood storage at ambient temperature (Fig. 5b). The predicted size of intact TSP-1 is 133 kDa. However, PROTOMAP and immunoblotting revealed a storage time-dependent accumulation of protein species in the range of 120–150 kDa, most likely due to increased cellular secretion and proteolytic processing. C4B complement protein was identified in plasma at ~200 kDa with predominant fragments at 100 and 75 kDa corresponding to α chain and β chain, respectively [26]. Interestingly, as indicated by the peptograph analysis, fragments observed in the range of 100–75 kDa were decreased in the 48 h plasma samples when compared to 30 min control samples, possibly as a result of secretion of newly expressed protein and subsequent enzymatic activity of the complement cascade that continues to occur during whole blood storage. Consistent with this, we observed an increased abundance of lower molecular weight fragments and the generation of novel fragments below the 50 kDa mark over time (Fig. 6). Notably, a cluster of fragments between 35–40 kDa potentially corresponding to the C4d complement protein [26] was gradually increased with storage time. The C4B peptograph indicates the generation of two more C4B derived fragments at ~30 kDa that might correspond to C4 γ chain and an additional fragment at ~15 kDa [26], which were not detected by immunoblotting. PROTOMAP analysis also detected similar proteolytic degradation patterns of other complement proteins such as C1r, C2, C3 and C5 (Figs. 4, Additional file 1: S1). Combined, these results indicate partial proteolytic processing of a small sub-fraction of members of the complement cascade upon blood storage at ambient temperature.

## Discussion

Blood is the commonest clinical sample collected in biobanks. We embarked on this study to optimise the quality of clinical samples whilst establishing a UK Biobank in organ donation and transplantation (QUOD) collecting biomaterial from more than 60 hospital sites including out-of-hours and in week-ends [4]. Despite a general tendency in bio-banking to store whole blood in hypothermic temperatures of around 4 °C prior to sample processing, we favoured whole blood samples to remain at ambient temperature prior to plasma preparation. Our rationale was to minimize platelet and leucocyte activation and the subsequent release of cytosolic proteins and enzymes that could constitute a potential source of bias [12]. Previously, we assessed how the plasma proteome is altered while whole blood stored in AT and can introduce a bias on selection of candidate proteins in biomarker discovery studies. Remarkably, under these conditions, less than 5 % of the identified 430 proteins were found to be altered [18].

The limited impact of ambient temperature in plasma proteome dynamics is consistent with the study by Aguilar-Mahecha and colleagues reporting minimal variability on medium to high abundant plasma proteins when whole blood remained at ambient temperature for up to 6 h as opposed to when stored and processed at 4 °C [27]. Also, no benefit in protein stability was found when blood tubes containing proteinase inhibitors were used for whole blood remaining at ambient temperature up to 6 h. Similarly, when we analysed naturally occurring peptides in plasma by LC–MS/MS, we only identified fragments from a small number of proteins, even after 48 h storage (Fig. 3d).

Proteolysis is an intrinsic homeostatic phenomenon in blood, and for the consideration of proteolytic protein fragments to be potential biomarkers [28], we investigated the degree of plasma proteolysis due to collection and storage conditions. The plasma peptidome (derived from protein degradation) has been a source of novel biomarkers for human diseases including transplantation [29–32]. We used a global proteolytic topography and migration analysis platform (PROTOMAP) to assess plasma proteolysis. Two extreme conditions, 30 min and 48 h, were selected to demonstrate the full effect of proteolysis, reflecting blood specimens collected in the clinic and remaining at ambient temperature over prolonged times or weekends prior to diagnostic analysis. Next to providing insights into proteolysis dynamics, SDS-PAGE based fractionation prior to LC–MS/MS analysis extended the number of plasma protein identification to 820. For 20 proteins, we observed enrichment and the appearance of protein isoforms that were uniquely identified in the 48 h pool, and 22 proteins exerted a clear proteolysis profile. Thrombospondin-1 (TSP-1) was identified in both, single LC–MS/MS and PROTOMAP analysis (Fig. 5a, b). A gradual increase in TSP-1 levels was observed between 30 min, 8, 24 and 48 h and also confirmed by immunoblotting (Fig. 5). TSP-1, a 450 kDa matricellular protein, is one of the main constituents of alpha granules released from platelets following activation. Upon release, TSP-1 undergoes proteolysis to generate several fragments in the 140-, 75-, 50- and 25 kDa range [33]. Interestingly, some of these fragments appear to overlap with degradation intermediates of TSP1 that have anti-angiogenic properties [34]. PROTOMAP analysis also revealed an increase in a 150 kDa fragment with additional fragments in the range of 50–150 kDa. Notably, a 28 kDa N-terminal fragment was found to be moderately enriched in the 48 h condition (Fig. 5a). This N-terminal domain may be a heparin binding domain that derives from serine-dependent proteolysis within the platelet α granules [33–36]. This heparin binding fragment subsequently migrates to the platelet membrane to interact with the actin cytoskeleton and fibrin to stabilise platelet aggregates during platelet activation. It is currently unknown whether the release of this fragment was the result of degranulation due a low degree of platelet activation or whether it has been shed in plasma from platelet membranes. Changes in circulating levels of TSP-1 fragments have been considered as potential biomarkers of tumour cell metastasis, inflammation, haemostasis and thrombosis [37–40]. Pre-analytical variability of TSP-1 as described here can provide a baseline for more accurate diagnostic detection in plasma samples.

PROTOMAP also detected proteolytic fragments of the complement proteins C1r, C3, C4B and C5 (Fig. 6, Additional file 1: S1). Activation of platelets, even to a low degree, can activate and propagate the complement system [41]. Similarly, the circulation of complement fragments in plasma can trigger platelet activation. Potential triggers of platelet activation can be particles that are released or shed from white cells such as neutrophils or simply from the sheer stress to blood extracted from the vascular system during collection.

In summary, our study revealed remarkably few changes in plasma proteome dynamics with some distinct cases of proteins undergoing proteolytic degradation which affects their usefulness as disease biomarkers. More generally, blood sample storage at ambient temperature appears suitable for maintaining high quality in the clinical context, in particular at highly unconventional hours of collection occurring in many hospital sites.

## Additional file

10.1186/s12014-016-9126-9 Blood plasma proteins identified and quantified by mass spectrometry. **Figure S1.** Peptographs of complement proteins C2 and C5 (*red bars*: 30 min; *blue bars*: 48 h). **Figure S2.** Peptographs of additional proteins (*red bars*: 30 min; *blue bars*: 48 h).

## Abbreviations

PROTOMAP: protein topography and migration analysis platform
LC–MS/MS: liquid chromatography tandem mass spectrometry
AT: ambient temperature
rpm: rotations per minute
TSP-1: thrombosbondin-1
C4: complement C4
QUOD: quality in organ donation
