# Supplementary material for: Plasma degradome affected by variable storage of human blood
Source: Clin Proteomics. 2016 Sep 26;13:26. doi: 10.1186/s12014-016-9126-9 (PMC5037888; doi:10.1186/s12014-016-9126-9)
Supplement: Supplementary file 1 — 10.1186/s12014-016-9126-9 Blood plasma proteins identified and quantified by mass spectrometry. Figure S1. Peptographs of complement proteins C2 and C5 (red bars: 30 min; blue bars: 48 h). Figure S2. Peptographs of additional proteins (red bars: 30 min; blue bars: 48 h). [file 12014_2016_9126_MOESM1_ESM.pdf]

Plasma Degradome Affected by Variable Storage of Human Blood

Maria Kaisar[1,2,4], Leon F.A. van Dullemen[3+], Marie-Laëtitia Thézénas[4+], M. Zeeshan Akhtar[1], Honglei Huang[1,4], Sandrine Rendel[1], Philip D. Charles[4], Roman Fischer[4], Rutger J. Ploeg[1,2§] and Benedikt M. Kessler[4§]

[1] *Nuffield Department of Surgical Sciences, University of Oxford, OX3 7LJ, UK*
[2]*NHS Blood and Transplant, UK*
[3]*Surgical Research Laboratory, University of Groningen, The Netherlands*
[4]*Target Discovery Institute, Nuffield Department of Medicine, University of Oxford, OX3 7FZ, UK*

+Authors have contributed equally
§Authors have contributed equally

Corresponding Author: Benedikt M Kessler
Target Discovery Institute,
Nuffield Department of Medicine,
University of Oxford,
OX3 7FZ, UK
Email: Benedikt.Kessler@ndm.ox.ac.uk

Supplemental Data

Table S1

Figure S1

Figure S2

Figure Legend

Figure S1: ***Peptographs of Complement proteins indicating partial degradation as a function of variable blood storage***

Prolonged blood storage provokes partial degradation of complement C5 **(A)** and C2 **(B)** as exemplified by their corresponding peptographs (blue bars – 30 min; red bars – 48 h blood storage at ambient temperature).

Figure S2: ***Peptographs indicate partial degradation* of *plasma proteins as a function of variable blood storage***

Prolonged blood storage provokes partial degradation of fibrinogen alpha chain, serpin A4 (corticosteroid-binding globulin), serpin A3 (serum paraoxonase), extracellular matrix protein-1 (EMC1), ceruloplasmin, plasminogen like protein A, serum paraoxonase (PON1), alpha-1 inter alpha trypsin inhibitor (ITIH1), fibronectin and apolipoprotein B-100 as exemplified by their corresponding peptographs (blue bars – 30 min; red bars – 48 h blood storage at ambient temperature).

Table S1

			Average normalised abundance				Fold Change		
Accession	Description	Anova (p)*	Centrifugation T=30 min	Centrifugation T=8h	Centrifugation T=24h	Centrifugation T=48h	8h vs 30m	24h vs 30min	48h vs 30m
Q4G0Z9	MCM domain-containing protein 2	8.30E-07	205000	332000	342000	347000	1.62	1.67	1.69
P02768	Serum albumin	1.01E-05	10100000	17900000	19000000	18300000	1.77	1.88	1.81
Q07864	DNA polymerase epsilon catalytic subunit A	1.28E-05	628000	783000	780000	741000	1.25	1.24	1.18
P07737	Profilin-1	2.86E-05	12900	14300	25900	92200	1.11	2.01	7.15
Q96N67	Dedicator of cytokinesis protein 7	6.08E-04	1030000	1530000	1530000	1430000	1.49	1.49	1.39
Q96M91	Coiled-coil domain-containing protein 11	8.62E-04	744000	902000	974000	932000	1.21	1.31	1.25
P0CG06	Ig lambda-3 chain C regions	1.25E-03	88300	159000	166000	172000	1.80	1.88	1.95
P07996	Thrombospondin-1	1.41E-03	449000	537000	601000	625000	1.20	1.34	1.39
P02538	Keratin, type II cytoskeletal 6A	2.00E-03	900000	1100000	1130000	1120000	1.22	1.26	1.24
P02746	Complement C1q subcomponent subunit B	2.50E-03	1660000	2160000	2140000	2110000	1.30	1.29	1.27
Q53SZ7	Uncharacterized protein C2orf53	2.57E-03	262000	480000	495000	454000	1.83	1.89	1.73
P01620	Ig kappa chain V-III region SIE;	8.92E-03	2729.32	5438.74	5586.37	6118.34	1.99	2.05	2.24
P01857	Ig gamma-1 chain C region;	1.00E-02	324000	578000	596000	575000	1.78	1.84	1.77
P02647	Apolipoprotein A-I	2.00E-02	434000	553000	553000	559000	1.27	1.27	1.29
P01860	Ig gamma-3 chain C region	2.00E-02	187000	316000	322000	318000	1.69	1.72	1.70
P06326	Ig heavy chain V-I region Mot	2.00E-02	3822.83	6588.17	7772.15	7257.39	1.72	2.03	1.90
Q6NXT1	Ankyrin repeat domain-containing protein 54	2.00E-02	57400	128000	130000	124000	2.23	2.26	2.16
P05160	Coagulation factor XIII B chain	3.00E-02	965000	1200000	1180000	1170000	1.24	1.22	1.21
O75019	Leukocyte immunoglobulin-like receptor subfamily A member 1	3.00E-02	54300	67700	76500	73800	1.25	1.41	1.36
P02679	Fibrinogen gamma chain	4.00E-02	1710000	2350000	2220000	2120000	1.37	1.30	1.24
P01876	Ig alpha-1 chain C region;	4.00E-02	34000	83100	84000	78100	2.44	2.47	2.30
P01034	Cystatin-C	4.00E-02	38600	50800	49800	48900	1.32	1.29	1.27
P01834	Ig kappa chain C region;	5.00E-02	203000	412000	437000	421000	2.03	2.15	2.07
P11021	78 kDa glucose-regulated protein	5.00E-02	1010000	1290000	1270000	1230000	1.28	1.26	1.22
Q8IUH4	Palmitoyltransferase ZDHHC13	5.00E-02	1730000	1500000	1580000	1490000	0.87	0.91	0.86
P01619	Ig kappa chain V-III region B6	6.00E-02	2022.75	3676	3845.89	4375.64	1.82	1.90	2.16
P00738	Haptoglobin	8.00E-02	133000	209000	220000	214000	1.57	1.65	1.61
P02775	Platelet basic protein	8.00E-02	199000	177000	285000	322000	0.89	1.43	1.62
P07951	Tropomyosin beta chain	8.00E-02	700000	851000	940000	943000	1.22	1.34	1.35
P06753	Tropomyosin alpha-3 chain	8.00E-02	705000	858000	938000	931000	1.22	1.33	1.32
P54108	Cysteine-rich secretory protein 3	9.00E-02	73400	95700	102000	99900	1.30	1.39	1.36
Q86U86	Protein polybromo-1;	1.00E-01	3080000	3550000	3630000	3610000	1.15	1.18	1.17
P02776	Platelet factor 4; S	1.00E-01	8573.19	7584.43	13700	21300	0.88	1.60	2.48
P31949	Protein S100-A11	1.20E-01	14.45	0	274.09	574.75	0.00	18.97	39.78
P01859	Ig gamma-2 chain C region;	1.50E-01	262000	405000	389000	380000	1.55	1.48	1.45
P06702	Protein S100-A9	1.60E-01	17300	19100	96400	136000	1.10	5.57	7.86
P01861	Ig gamma-4 chain C region;	1.70E-01	411000	512000	544000	516000	1.25	1.32	1.26
P00558	Phosphoglycerate kinase 1	1.70E-01	3400000	3070000	3190000	3030000	0.90	0.94	0.89
Q16322	Potassium voltage-gated channel subfamily A member 10	1.70E-01	132000	103000	97800	99300	0.78	0.74	0.75
P00736	Complement C1r subcomponent	1.80E-01	3060000	2740000	2790000	2610000	0.90	0.91	0.85
O15481	Melanoma-associated antigen B4	2.00E-01	822000	712000	765000	741000	0.87	0.93	0.90
Q8IWN7	Retinitis pigmentosa 1-like 1 protein;	2.30E-01	1750000	1900000	2020000	1990000	1.09	1.15	1.14
Q14532	Keratin, type I cuticular Ha2	2.30E-01	1500000	1560000	1760000	1780000	1.04	1.17	1.19
Q12860	Contactin-1; AltName	2.30E-01	68700	56200	66100	65500	0.82	0.96	0.95
P01009	Alpha-1-antitrypsin	2.40E-01	387000	405000	436000	413000	1.05	1.13	1.07
O75882	Attractin	2.70E-01	995000	896000	843000	825000	0.90	0.85	0.83
P03951	Coagulation factor XI	3.00E-01	433000	392000	406000	402000	0.91	0.94	0.93
P05109	Protein S100-A8	3.00E-01	34700	31400	57700	80800	0.90	1.66	2.33
Q5T013	Putative hydroxypyruvate isomerase	3.00E-01	1090000	1260000	1140000	1120000	1.16	1.05	1.03
P14618	Pyruvate kinase PKM	3.00E-01	342000	304000	317000	324000	0.89	0.93	0.95
P35908	Keratin, type II cytoskeletal 2 epidermal	3.10E-01	565000	495000	498000	474000	0.88	0.88	0.84
P00915	Carbonic anhydrase 1	3.40E-01	1670000	1900000	1840000	1750000	1.14	1.10	1.05
Q6TDU7	Protein CASC1	3.80E-01	706000	644000	610000	558000	0.91	0.86	0.79
Q02985	Complement factor H-related protein 3	4.10E-01	519000	508000	460000	459000	0.98	0.89	0.88
P50406	5-hydroxytryptamine receptor 6	4.10E-01	139000	142000	117000	112000	1.02	0.84	0.81
Q8IXR9	Uncharacterized protein C12orf56	4.30E-01	982000	1110000	1130000	1020000	1.13	1.15	1.04
P25815	Protein S100-P	4.50E-01	10300	10500	13100	13600	1.02	1.27	1.32
P02787	Serotransferrin	4.60E-01	854000	963000	973000	922000	1.13	1.14	1.08
Q9NQ79	Cartilage acidic protein 1	4.60E-01	13500	12100	15600	15600	0.90	1.16	1.16
Q13103	Secreted phosphoprotein 24	4.70E-01	147000	132000	118000	111000	0.90	0.80	0.76
Q14168	MAGUK p55 subfamily member 2	4.70E-01	610000	648000	662000	665000	1.06	1.09	1.09
Q9H939	Proline-serine-threonine phosphatase-interacting protein 2	4.70E-01	1290000	1210000	1350000	1350000	0.94	1.05	1.05
Q9NQI0	Probable ATP-dependent RNA helicase DDX4	4.70E-01	926000	1060000	1010000	903000	1.14	1.09	0.98
Q9NR34	Mannosyl-oligosaccharide 1,2-alpha-mannosidase IC	4.70E-01	103000	85900	105000	102000	0.83	1.02	0.99
Q04756	Hepatocyte growth factor activator	4.80E-01	1070000	939000	1020000	998000	0.88	0.95	0.93
P15923	Transcription factor E2-alpha	4.80E-01	738000	851000	797000	697000	1.15	1.08	0.94
P06396	Gelsolin	4.90E-01	10500000	9650000	10100000	9960000	0.92	0.96	0.95
Q9Y490	Talin-1	4.90E-01	5690000	5240000	5300000	5340000	0.92	0.93	0.94
Q6YHU6	Thyroid adenoma-associated protein	4.90E-01	4720000	4840000	5220000	5140000	1.03	1.11	1.09
Q6UVK1	Chondroitin sulfate proteoglycan 4	4.90E-01	1710000	1650000	1720000	1600000	0.96	1.01	0.94
P17936	Insulin-like growth factor-binding protein 3	5.00E-01	143000	125000	136000	131000	0.87	0.95	0.92
Q5VZM2	Ras-related GTP-binding protein B	5.00E-01	374000	313000	370000	353000	0.84	0.99	0.94
P02654	Apolipoprotein C-I	5.20E-01	286000	234000	243000	326000	0.82	0.85	1.14
P48741	Putative heat shock 70 kDa protein 7	5.20E-01	999000	915000	925000	898000	0.92	0.93	0.90
P02753	Retinol-binding protein 4	5.30E-01	2800000	3030000	3330000	2780000	1.08	1.19	0.99
P00746	Complement factor D	5.30E-01	57500	50900	53900	56100	0.89	0.94	0.98
P23142	Fibulin-1	5.40E-01	689000	632000	710000	736000	0.92	1.03	1.07

Q8IZJ4	Ral-GDS-related protein	5.40E-01	608000	484000	546000	613000	0.80	0.90	1.01
P12259	Coagulation factor V	5.60E-01	7530000	6920000	7020000	6750000	0.92	0.93	0.90
Q9Y2V7	Conserved oligomeric Golgi complex subunit 6	5.60E-01	930000	844000	872000	834000	0.91	0.94	0.90
P61626	Lysozyme C	5.70E-01	106000	98000	94800	106000	0.92	0.89	1.00
P55056	Apolipoprotein C-IV	5.70E-01	57000	51700	51200	50200	0.91	0.90	0.88
Q9Y2K3	Myosin-15	5.80E-01	6180000	5810000	5860000	5740000	0.94	0.95	0.93
P0DJI8	Serum amyloid A-1 protein	5.80E-01	4916.99	6706.49	9214.87	14700	1.36	1.87	2.99
P63104	14-3-3 protein zeta/delta	5.80E-01	101000	86700	81600	90600	0.86	0.81	0.90
Q6ZTQ3	Ras association domain-containing protein 6	5.80E-01	2230000	2070000	2290000	2110000	0.93	1.03	0.95
P04220	Ig mu heavy chain disease protein	5.90E-01	33500	51000	48500	45500	1.52	1.45	1.36
O94901	SUN domain-containing protein 1	5.90E-01	5210000	4930000	4750000	4680000	0.95	0.91	0.90
Q03591	Complement factor H-related protein 1	6.00E-01	2340000	2150000	2150000	2200000	0.92	0.92	0.94
Q9H8V3	Protein ECT2	6.00E-01	1820000	1670000	1610000	1610000	0.92	0.88	0.88
P02790	Hemopexin	6.10E-01	50200000	47300000	48100000	46600000	0.94	0.96	0.93
P37802	Transgelin-2	6.10E-01	2380000	2590000	2610000	2480000	1.09	1.10	1.04
P23528	Cofilin-1	6.10E-01	266000	238000	247000	260000	0.89	0.93	0.98
P00338	L-lactate dehydrogenase A chain	6.10E-01	1230000	1110000	1100000	1070000	0.90	0.89	0.87
Q8IZF0	Sodium leak channel non-selective protein	6.20E-01	1150000	1070000	1080000	1060000	0.93	0.94	0.92
O75626	PR domain zinc finger protein 1	6.20E-01	477000	398000	390000	381000	0.83	0.82	0.80
P02649	Apolipoprotein E	6.30E-01	3660000	3560000	3590000	3330000	0.97	0.98	0.91
Q15195	Plasminogen-like protein A	6.30E-01	238000	214000	226000	225000	0.90	0.95	0.95
Q96PV7	Protein FAM193B;	6.30E-01	283000	240000	257000	276000	0.85	0.91	0.98
Q9BYX7	Putative beta-actin-like protein 3	6.40E-01	464000	411000	426000	462000	0.89	0.92	1.00
P02766	Transthyretin	6.50E-01	110000	125000	120000	96800	1.14	1.09	0.88
P07195	L-lactate dehydrogenase B chain	6.50E-01	211000	197000	205000	218000	0.93	0.97	1.03
P02745	Complement C1q subcomponent subunit A	6.60E-01	719000	808000	769000	744000	1.12	1.07	1.03
O00391	Sulfhydryl oxidase 1	6.60E-01	4450000	4070000	3970000	3860000	0.91	0.89	0.87
Q8WVM7	Cohesin subunit SA-1	6.60E-01	2500000	2250000	2330000	2430000	0.90	0.93	0.97
P04217	Alpha-1B-glycoprotein	6.70E-01	9040000	8660000	8780000	8380000	0.96	0.97	0.93
P05154	Plasma serine protease inhibitor	6.70E-01	138000	115000	122000	152000	0.83	0.88	1.10
P00742	Coagulation factor X	6.70E-01	946000	1030000	992000	953000	1.09	1.05	1.01
P43121	Cell surface glycoprotein MUC18	6.70E-01	3310000	2870000	3070000	3010000	0.87	0.93	0.91
Q96CF2	Charged multivesicular body protein 4c	6.70E-01	375000	361000	359000	335000	0.96	0.96	0.89
P02765	Alpha-2-HS-glycoprotein	6.80E-01	24200000	22200000	23300000	22000000	0.92	0.96	0.91
Q04695	Keratin, type I cytoskeletal 17	6.80E-01	1760000	1590000	1770000	1620000	0.90	1.01	0.92
Q9Y5Y7	Lymphatic vessel endothelial hyaluronic acid receptor 1; Short=LYV	6.80E-01	6220000	6560000	6680000	6820000	1.05	1.07	1.10
P01011	Alpha-1-antichymotrypsin	6.90E-01	10600000	10200000	10100000	9790000	0.96	0.95	0.92
Q9UK55	Protein Z-dependent protease inhibitor	6.90E-01	6220000	6560000	6670000	6810000	1.05	1.07	1.09
P01877	Ig alpha-2 chain C region	6.90E-01	200000	227000	230000	224000	1.14	1.15	1.12
P02763	Alpha-1-acid glycoprotein 1	6.90E-01	110000	136000	111000	105000	1.24	1.01	0.95
P19652	Alpha-1-acid glycoprotein 2	6.90E-01	115000	146000	123000	115000	1.27	1.07	1.00
Q96AY3	Peptidyl-prolyl cis-trans isomerase FKBP10	6.90E-01	420000	407000	388000	357000	0.97	0.92	0.85
P07360	Complement component C8 gamma chain	7.00E-01	839000	761000	801000	773000	0.91	0.95	0.92
Q9Y4G6	Talin-2	7.00E-01	4250000	3840000	4050000	4060000	0.90	0.95	0.96
Q38SD2	Leucine-rich repeat serine/threonine-protein kinase 1	7.00E-01	4570000	4160000	4070000	3970000	0.91	0.89	0.87
Q15166	Serum paraoxonase/lactonase 3	7.00E-01	183000	193000	195000	206000	1.05	1.07	1.13
P04040	Catalase	7.00E-01	3170000	2950000	3060000	2930000	0.93	0.97	0.92
Q8IYB4	PEX5-related protein	7.00E-01	1320000	1320000	1430000	1480000	1.00	1.08	1.12
O75636	Ficolin-3	7.10E-01	382000	326000	340000	349000	0.85	0.89	0.91
Q92954	Proteoglycan 4	7.10E-01	5420000	5080000	5220000	4960000	0.94	0.96	0.92
Q92817	Envoplakin	7.10E-01	7280000	7300000	7250000	6900000	1.00	1.00	0.95
Q00610	Clathrin heavy chain 1	7.10E-01	4530000	4430000	4290000	4180000	0.98	0.95	0.92
P02751	Fibronectin	7.20E-01	515000	474000	495000	474000	0.92	0.96	0.92
P04264	Keratin, type II cytoskeletal 1	7.20E-01	524000	489000	493000	443000	0.93	0.94	0.85
P36980	Complement factor H-related protein 2	7.20E-01	1100000	974000	1010000	1020000	0.89	0.92	0.93
O60524	Nuclear export mediator factor NEMF	7.20E-01	2420000	2090000	2350000	2390000	0.86	0.97	0.99
P57103	Sodium/calcium exchanger 3	7.20E-01	793000	737000	685000	716000	0.93	0.86	0.90
P20366	Protachykinin-1	7.20E-01	61900	52700	56300	55600	0.85	0.91	0.90
P09172	Dopamine beta-hydroxylase	7.30E-01	318000	290000	291000	313000	0.91	0.92	0.98
P04259	Keratin, type II cytoskeletal 6B	7.30E-01	1250000	1310000	1210000	1270000	1.05	0.97	1.02
P08709	Coagulation factor VII	7.30E-01	423000	405000	367000	366000	0.96	0.87	0.87
Q6ZN30	Zinc finger protein basonuclin-2	7.30E-01	1460000	1320000	1390000	1310000	0.90	0.95	0.90
Q6P9F7	Leucine-rich repeat-containing protein 8B	7.30E-01	307000	302000	293000	286000	0.98	0.95	0.93
P27487	Dipeptidyl peptidase 4	7.30E-01	1180000	1080000	1100000	1150000	0.92	0.93	0.97
Q9Y5Z7	Host cell factor 2	7.40E-01	993000	1020000	1100000	1010000	1.03	1.11	1.02
P20929	Nebulin	7.50E-01	31900000	30500000	30700000	29700000	0.96	0.96	0.93
P02656	Apolipoprotein C-III	7.50E-01	1070000	989000	954000	1120000	0.92	0.89	1.05
P0DJI9	Serum amyloid A-2 protein	7.50E-01	43400	40700	39700	45200	0.94	0.91	1.04
Q9Y6K5	2'-5'-oligoadenylate synthase 3	7.50E-01	511000	508000	521000	549000	0.99	1.02	1.07
Q7Z3Y7	Keratin, type I cytoskeletal 28	7.50E-01	2070000	2000000	1990000	1910000	0.97	0.96	0.92
Q9UHC7	E3 ubiquitin-protein ligase makorin-1	7.50E-01	526000	475000	539000	509000	0.90	1.02	0.97
Q92769	Histone deacetylase 2	7.50E-01	2200000	2020000	1930000	1890000	0.92	0.88	0.86
P09871	Complement C1s subcomponent	7.60E-01	2950000	2690000	2880000	2820000	0.91	0.98	0.96
P26927	Hepatocyte growth factor-like protein	7.60E-01	924000	842000	851000	827000	0.91	0.92	0.90
P15169	Carboxypeptidase N catalytic chain	7.60E-01	2140000	1790000	1980000	1980000	0.84	0.93	0.93
Q92820	Gamma-glutamyl hydrolase	7.60E-01	1240000	1260000	1310000	1200000	1.02	1.06	0.97
Q14515	SPARC-like protein 1	7.60E-01	787000	881000	783000	772000	1.12	0.99	0.98
Q9UNP4	Lactosylceramide alpha-2,3-sialyltransferase	7.60E-01	11000	9538.5	9976.91	10000	0.87	0.91	0.91
P68032	Actin, alpha cardiac muscle 1	7.70E-01	534000	493000	485000	564000	0.92	0.91	1.06
Q8NDH2	Coiled-coil domain-containing protein 168	7.70E-01	10600000	10300000	10100000	9960000	0.97	0.95	0.94

A8MT79	Putative zinc-alpha-2-glycoprotein-like 1	7.70E-01	701000	636000	680000	661000	0.91	0.97	0.94
P08107	Heat shock 70 kDa protein 1A/1B	7.70E-01	1720000	1780000	1910000	1750000	1.03	1.11	1.02
O15014	Zinc finger protein 609	7.70E-01	1400000	1370000	1340000	1310000	0.98	0.96	0.94
P19823	Inter-alpha-trypsin inhibitor heavy chain H2	7.80E-01	16800000	15200000	16300000	15600000	0.90	0.97	0.93
Q9NZP8	Complement C1r subcomponent-like protein	7.80E-01	180000	157000	171000	158000	0.87	0.95	0.88
O14791	Apolipoprotein L1	7.80E-01	258000	247000	245000	247000	0.96	0.95	0.96
Q9Y2I6	Ninein-like protein	7.80E-01	1270000	1200000	1280000	1260000	0.94	1.01	0.99
Q9H330	Transmembrane protein 245	7.80E-01	620000	622000	603000	574000	1.00	0.97	0.93
P26599	Polypyrimidine tract-binding protein 1	7.80E-01	491000	468000	471000	451000	0.95	0.96	0.92
P01008	Antithrombin-III	7.90E-01	13200000	12300000	12600000	12300000	0.93	0.95	0.93
Q12805	EGF-containing fibulin-like extracellular matrix protein 1	7.90E-01	218000	218000	235000	221000	1.00	1.08	1.01
Q2TV78	Putative macrophage stimulating 1-like protein	7.90E-01	211000	202000	197000	189000	0.96	0.93	0.90
P31146	Coronin-1A	7.90E-01	1090000	970000	1070000	1110000	0.89	0.98	1.02
P05019	Insulin-like growth factor II	7.90E-01	468000	438000	444000	436000	0.94	0.95	0.93
Q9BYJ9	YTH domain family protein 1	7.90E-01	2020000	1870000	1860000	1830000	0.93	0.92	0.91
P0C0L5	Complement C4-B	8.00E-01	27100000	26600000	25500000	24600000	0.98	0.94	0.91
P02760	Protein AMBP	8.00E-01	3700000	3410000	3550000	3460000	0.92	0.96	0.94
P04004	Vitronectin	8.00E-01	8040000	7730000	7800000	7540000	0.96	0.97	0.94
P01766	Ig heavy chain V-III region BRO;	8.00E-01	367000	393000	392000	368000	1.07	1.07	1.00
Q8IZF3	Probable G-protein coupled receptor 115	8.00E-01	1010000	987000	939000	941000	0.98	0.93	0.93
P22314	Ubiquitin-like modifier-activating enzyme 1	8.00E-01	550000	569000	542000	526000	1.03	0.99	0.96
P0C0L4	Complement C4-A	8.10E-01	26700000	26200000	25200000	24300000	0.98	0.94	0.91
P04003	C4b-binding protein alpha chain	8.10E-01	459000	403000	387000	465000	0.88	0.84	1.01
Q9BXR6	Complement factor H-related protein 5	8.10E-01	956000	958000	932000	877000	1.00	0.97	0.92
P01871	Ig mu chain C region	8.10E-01	425000	353000	385000	399000	0.83	0.91	0.94
Q8NI35	InaD-like protein	8.10E-01	2920000	2880000	2920000	3090000	0.99	1.00	1.06
Q01518	Adenylyl cyclase-associated protein 1	8.10E-01	1520000	1570000	1510000	1450000	1.03	0.99	0.95
P49746	Thrombospondin-3	8.10E-01	2020000	2050000	2110000	2010000	1.01	1.04	1.00
P02671	Fibrinogen alpha chain	8.20E-01	2850000	2830000	2950000	3010000	0.99	1.04	1.06
P27169	Serum paraoxonase/arylesterase 1	8.20E-01	1130000	1230000	1250000	1270000	1.09	1.11	1.12
P61769	Beta-2-microglobulin	8.20E-01	102000	97500	107000	103000	0.96	1.05	1.01
O60287	Nucleolar pre-ribosomal-associated protein 1	8.20E-01	415000	425000	443000	436000	1.02	1.07	1.05
P62937	Peptidyl-prolyl cis-trans isomerase A	8.20E-01	158000	148000	166000	164000	0.94	1.05	1.04
Q70Z53	Protein FRA10AC1	8.20E-01	98300	80000	93300	97100	0.81	0.95	0.99
Q562R1	Beta-actin-like protein 2	8.30E-01	277000	256000	255000	303000	0.92	0.92	1.09
Q03164	Histone-lysine N-methyltransferase 2A	8.30E-01	4130000	3960000	3950000	3940000	0.96	0.96	0.95
P14151	L-selectin	8.30E-01	193000	181000	168000	174000	0.94	0.87	0.90
Q9BYU1	Pre-B-cell leukemia transcription factor 4	8.30E-01	4450000	4280000	4140000	4020000	0.96	0.93	0.90
B5MCN3	Putative SEC14-like protein 6	8.30E-01	1960000	1810000	1840000	1890000	0.92	0.94	0.96
Q99542	Matrix metalloproteinase-19	8.30E-01	683000	626000	665000	657000	0.92	0.97	0.96
Q9HC29	Nucleotide-binding oligomerization domain-containing protein 2	8.30E-01	1360000	1480000	1410000	1470000	1.09	1.04	1.08
O43692	Peptidase inhibitor 15	8.30E-01	48100	41800	45000	48200	0.87	0.94	1.00
Q5SYB0	FERM and PDZ domain-containing protein 1	8.40E-01	1170000	1220000	1150000	1150000	1.04	0.98	0.98
P0C091	FRAS1-related extracellular matrix protein 3	8.40E-01	4320000	4320000	4380000	4060000	1.00	1.01	0.94
P17014	Zinc finger protein 12	8.40E-01	348000	382000	382000	391000	1.10	1.10	1.12
Q6DD88	Atlastin-3	8.40E-01	1020000	954000	945000	964000	0.94	0.93	0.95
A5YKK6	CCR4-NOT transcription complex subunit 1	8.50E-01	7660000	7940000	8050000	8170000	1.04	1.05	1.07
Q8WXW3	Progesterone-induced-blocking factor 1	8.50E-01	2020000	1910000	2010000	1960000	0.95	1.00	0.97
Q92905	COP9 signalosome complex subunit 5	8.50E-01	972000	967000	962000	901000	0.99	0.99	0.93
Q9H4L7	SWI/SNF-related matrix-associated actin-dependent regulator of c	8.50E-01	1360000	1300000	1310000	1350000	0.96	0.96	0.99
O94760	N(G),N(G)-dimethylarginine dimethylaminohydrolase 1	8.50E-01	4860000	4630000	4550000	4450000	0.95	0.94	0.92
Q9Y5K1	Meiotic recombination protein SPO11	8.50E-01	5150000	4890000	5180000	4950000	0.95	1.01	0.96
P29622	Kallistatin	8.60E-01	749000	701000	712000	700000	0.94	0.95	0.93
P18428	Lipopolysaccharide-binding protein	8.60E-01	132000	111000	121000	125000	0.84	0.92	0.95
O43866	CD5 antigen-like	8.60E-01	1980000	2110000	2010000	1850000	1.07	1.02	0.93
P04406	Glyceraldehyde-3-phosphate dehydrogenase	8.60E-01	52600	48500	49200	55400	0.92	0.94	1.05
Q9UL68	Myelin transcription factor 1-like protein	8.60E-01	6400000	6490000	6640000	6630000	1.01	1.04	1.04
O95445	Apolipoprotein M	8.60E-01	147000	137000	143000	155000	0.93	0.97	1.05
P35900	Keratin, type I cytoskeletal 20	8.60E-01	609000	600000	588000	599000	0.99	0.97	0.98
Q6ZNJ1	Neurobeachin-like protein 2	8.60E-01	1730000	1650000	1660000	1640000	0.95	0.96	0.95
A8MW99	Meiosis-specific protein MEI4-like	8.60E-01	2730000	2660000	2630000	2530000	0.97	0.96	0.93
P00748	Coagulation factor XII	8.70E-01	1900000	1670000	1760000	1710000	0.88	0.93	0.90
P04278	Sex hormone-binding globulin	8.70E-01	323000	349000	342000	321000	1.08	1.06	0.99
Q5VST9	Obscurin	8.70E-01	9920000	9270000	9500000	9450000	0.93	0.96	0.95
O75369	Filamin-B	8.70E-01	2370000	2220000	2380000	2190000	0.94	1.00	0.92
Q96BY6	Dedicator of cytokinesis protein 10	8.70E-01	2960000	3050000	2980000	2860000	1.03	1.01	0.97
P35542	Serum amyloid A-4 protein	8.70E-01	223000	231000	235000	245000	1.04	1.05	1.10
O43290	U4/U6.U5 tri-snRNP-associated protein 1	8.70E-01	608000	555000	571000	549000	0.91	0.94	0.90
P01614	Ig kappa chain V-II region Cum	8.70E-01	143000	157000	143000	128000	1.10	1.00	0.90
Q8WZA6	Olfactory receptor 1E3	8.70E-01	102000	110000	110000	101000	1.08	1.08	0.99
P01031	Complement C5	8.80E-01	7320000	7140000	7210000	7100000	0.98	0.98	0.97
P00734	Prothrombin	8.80E-01	11500000	11000000	11200000	10900000	0.96	0.97	0.95
P01042	Kininogen-1	8.80E-01	9600000	9170000	9460000	9390000	0.96	0.99	0.98
P36955	Pigment epithelium-derived factor	8.80E-01	1210000	1160000	1200000	1180000	0.96	0.99	0.98
P63261	Actin, cytoplasmic 2	8.80E-01	1590000	1480000	1470000	1580000	0.93	0.92	0.99
Q8NCM2	Potassium voltage-gated channel subfamily H member 5	8.80E-01	939000	959000	918000	911000	1.02	0.98	0.97
P35443	Thrombospondin-4	8.80E-01	2020000	1990000	2000000	2150000	0.99	0.99	1.06
Q9H892	Tetratricopeptide repeat protein 12	8.80E-01	1940000	1850000	1880000	1860000	0.95	0.97	0.96
P13671	Complement component C6	8.90E-01	3050000	2800000	2980000	2880000	0.92	0.98	0.94
P33151	Cadherin-5	8.90E-01	655000	653000	662000	630000	1.00	1.01	0.96
P04083	Annexin A1	8.90E-01	1610000	1550000	1600000	1570000	0.96	0.99	0.98
Q9BY43	Charged multivesicular body protein 4a	8.90E-01	294000	263000	283000	286000	0.89	0.96	0.97

Q99784	Noelin	8.90E-01	781000	711000	722000	694000	0.91	0.92	0.89
Q08380	Galectin-3-binding protein	8.90E-01	290000	243000	261000	250000	0.84	0.90	0.86
Q9NSB4	Keratin, type II cuticular Hb2	8.90E-01	54500	57100	51400	53300	1.05	0.94	0.98
Q7Z3D4	LysM and putative peptidoglycan-binding domain-containing prote	8.90E-01	622000	619000	638000	645000	1.00	1.03	1.04
P00740	Coagulation factor IX	9.00E-01	414000	440000	450000	434000	1.06	1.09	1.05
P02655	Apolipoprotein C-II	9.00E-01	505000	401000	388000	456000	0.79	0.77	0.90
Q15582	Transforming growth factor-beta-induced protein ig-h3	9.00E-01	847000	809000	798000	762000	0.96	0.94	0.90
P01344	Insulin-like growth factor II	9.00E-01	65000	66000	62800	60800	1.02	0.97	0.94
Q6PKG0	La-related protein 1	9.00E-01	1410000	1470000	1410000	1520000	1.04	1.00	1.08
Q16832	Discoidin domain-containing receptor 2	9.00E-01	1530000	1400000	1450000	1440000	0.92	0.95	0.94
Q9H221	ATP-binding cassette sub-family G member 8	9.00E-01	498000	505000	465000	485000	1.01	0.93	0.97
O75197	Low-density lipoprotein receptor-related protein 5	9.00E-01	389000	358000	375000	360000	0.92	0.96	0.93
A5A3E0	POTE ankyrin domain family member F	9.10E-01	2950000	2920000	3100000	3020000	0.99	1.05	1.02
Q6ZR08	Dynein heavy chain 12, axonemal	9.10E-01	5880000	5790000	5950000	5600000	0.98	1.01	0.95
P11226	Mannose-binding protein C	9.10E-01	531000	460000	500000	521000	0.87	0.94	0.98
P00451	Coagulation factor VIII	9.10E-01	6210000	6090000	6150000	6000000	0.98	0.99	0.97
Q9H6S0	Probable ATP-dependent RNA helicase YTHDC2	9.10E-01	3150000	3090000	3250000	3070000	0.98	1.03	0.97
P07205	Phosphoglycerate kinase 2	9.10E-01	1230000	1280000	1260000	1210000	1.04	1.02	0.98
P19013	Keratin, type II cytoskeletal 4	9.10E-01	1590000	1520000	1540000	1590000	0.96	0.97	1.00
Q9P2D3	HEAT repeat-containing protein 5B	9.10E-01	6490000	6170000	6450000	6280000	0.95	0.99	0.97
Q13586	Stromal interaction molecule 1	9.10E-01	1680000	1580000	1750000	1760000	0.94	1.04	1.05
O75674	TOM1-like protein 1	9.10E-01	527000	500000	526000	509000	0.95	1.00	0.97
A8MPT4	Glutathione S-transferase theta-4	9.10E-01	980000	958000	966000	941000	0.98	0.99	0.96
Q9H4A6	Golgi phosphoprotein 3	9.10E-01	401000	386000	404000	414000	0.96	1.01	1.03
P21333	Filamin-A	9.20E-01	5850000	5740000	5820000	5680000	0.98	0.99	0.97
P08571	Monocyte differentiation antigen CD14	9.20E-01	232000	247000	228000	234000	1.06	0.98	1.01
P78386	Keratin, type II cuticular Hb5	9.20E-01	1010000	901000	1000000	989000	0.89	0.99	0.98
O95497	Pantetheinase	9.20E-01	376000	390000	387000	366000	1.04	1.03	0.97
P04075	Fructose-bisphosphate aldolase A	9.20E-01	341000	318000	316000	318000	0.93	0.93	0.93
P09486	SPARC	9.20E-01	237000	234000	232000	243000	0.99	0.98	1.03
Q96A37	RING finger protein 166	9.20E-01	1040000	1040000	1010000	1010000	1.00	0.97	0.97
P08603	Complement factor H	9.30E-01	16200000	15500000	15600000	15700000	0.96	0.96	0.97
P07225	Vitamin K-dependent protein S	9.30E-01	981000	956000	945000	963000	0.97	0.96	0.98
P80108	Phosphatidylinositol-glycan-specific phospholipase D	9.30E-01	448000	490000	484000	463000	1.09	1.08	1.03
P02743	Serum amyloid P-component	9.30E-01	2920000	2780000	2780000	2780000	0.95	0.95	0.95
Q9Y6R7	IgGFc-binding protein	9.30E-01	1390000	1500000	1520000	1490000	1.08	1.09	1.07
Q02325	Plasminogen-like protein B	9.30E-01	482000	505000	513000	525000	1.05	1.06	1.09
P22891	Vitamin K-dependent protein Z	9.30E-01	39900	41300	40600	37300	1.04	1.02	0.93
P08727	Keratin, type I cytoskeletal 19	9.30E-01	689000	657000	679000	669000	0.95	0.99	0.97
Q9UIF8	Bromodomain adjacent to zinc finger domain protein 2B	9.30E-01	2600000	2740000	2730000	2690000	1.05	1.05	1.03
O00151	PDZ and LIM domain protein 1	9.30E-01	327000	348000	352000	330000	1.06	1.08	1.01
P22692	Insulin-like growth factor-binding protein 4	9.30E-01	13600	14400	13300	13700	1.06	0.98	1.01
P06276	Cholinesterase; EC=3.1.1.8	9.40E-01	2370000	2370000	2420000	2270000	1.00	1.02	0.96
P13796	Plastin-2	9.40E-01	571000	534000	534000	544000	0.94	0.94	0.95
P51587	Breast cancer type 2 susceptibility protein	9.40E-01	9920000	9690000	9850000	9530000	0.98	0.99	0.96
O00187	Mannan-binding lectin serine protease 2	9.40E-01	1380000	1300000	1380000	1330000	0.94	1.00	0.96
Q9P281	BAH and coiled-coil domain-containing protein 1	9.40E-01	5340000	5360000	5210000	5090000	1.00	0.98	0.95
Q6UXB8	Peptidase inhibitor 16	9.40E-01	265000	281000	268000	267000	1.06	1.01	1.01
P20851	C4b-binding protein beta chain	9.40E-01	67800	68100	69900	72700	1.00	1.03	1.07
P00918	Carbonic anhydrase 2	9.40E-01	854000	820000	806000	822000	0.96	0.94	0.96
Q9Y281	Cofilin-2	9.40E-01	652000	601000	584000	605000	0.92	0.90	0.93
P60174	Triosephosphate isomerase	9.40E-01	218000	205000	209000	210000	0.94	0.96	0.96
P22102	Trifunctional purine biosynthetic protein adenosine-3	9.40E-01	1160000	1160000	1100000	1100000	1.00	0.95	0.95
Q13330	Metastasis-associated protein MTA1;	9.40E-01	830000	878000	887000	857000	1.06	1.07	1.03
Q12799	T-complex protein 10A homolog;	9.40E-01	1660000	1720000	1740000	1650000	1.04	1.05	0.99
Q147U7	Single-pass membrane and coiled-coil domain-containing protein	9.40E-01	2480000	2250000	2320000	2180000	0.91	0.94	0.88
Q9NPY3	Complement component C1q receptor	9.40E-01	23600	24700	23000	24800	1.05	0.97	1.05
P19827	Inter-alpha-trypsin inhibitor heavy chain H1	9.50E-01	13500000	13200000	13100000	13000000	0.98	0.97	0.96
P03952	Plasma kallikrein; EC=3.4.21.34	9.50E-01	2220000	2110000	2140000	2130000	0.95	0.96	0.96
P07357	Complement component C8 alpha chain	9.50E-01	1850000	1820000	1910000	1860000	0.98	1.03	1.01
P18206	Vinculin	9.50E-01	9420000	9520000	9710000	9730000	1.01	1.03	1.03
Q7Z794	Keratin, type II cytoskeletal 1b	9.50E-01	1530000	1580000	1530000	1500000	1.03	1.00	0.98
O95678	Keratin, type II cytoskeletal 75	9.50E-01	1030000	1030000	1000000	1010000	1.00	0.97	0.98
O75366	Advillin	9.50E-01	640000	590000	556000	588000	0.92	0.87	0.92
Q6NW34	Uncharacterized protein C3orf17	9.50E-01	627000	672000	610000	598000	1.07	0.97	0.95
P02749	Beta-2-glycoprotein 1	9.60E-01	13400000	12700000	12600000	12300000	0.95	0.94	0.92
P10909	Clusterin	9.60E-01	5140000	5220000	5300000	5270000	1.02	1.03	1.03
P06681	Complement C2	9.60E-01	1840000	1790000	1780000	1730000	0.97	0.97	0.94
P0CG38	POTE ankyrin domain family member I	9.60E-01	2960000	2830000	2890000	2870000	0.96	0.98	0.97
Q86UX7	Fermitin family homolog 3	9.60E-01	79600	72300	77700	81400	0.91	0.98	1.02
A4D1E1	Zinc finger protein 804B	9.60E-01	2290000	2300000	2330000	2180000	1.00	1.02	0.95
P35442	Thrombospondin-2	9.60E-01	921000	908000	918000	875000	0.99	1.00	0.95
Q5T1M5	FK506-binding protein 15	9.60E-01	1750000	1710000	1720000	1780000	0.98	0.98	1.02
P01597	Ig kappa chain V-I region DEE	9.60E-01	35900	35100	35800	33200	0.98	1.00	0.92
Q6EMK4	Vasorin	9.60E-01	9240.63	8718.72	9390.51	9654.85	0.94	1.02	1.04
P12955	Xaa-Pro dipeptidase	9.60E-01	240000	217000	222000	225000	0.90	0.93	0.94
P61160	Actin-related protein 2	9.60E-01	311000	300000	302000	315000	0.96	0.97	1.01
P30043	Flavin reductase (NADPH)	9.60E-01	46600	48000	45500	48100	1.03	0.98	1.03
Q9BTU6	Phosphatidylinositol 4-kinase type 2-alpha	9.60E-01	116000	109000	115000	117000	0.94	0.99	1.01
P03950	Angiogenin	9.60E-01	8368.09	7940.01	7868.98	7822.79	0.95	0.94	0.93
Q8NBT3	Transmembrane protein 145	9.60E-01	38700	38100	37700	37200	0.98	0.97	0.96
P05546	Heparin cofactor 2	9.70E-01	1960000	1890000	1890000	1910000	0.96	0.96	0.97
P25311	Zinc-alpha-2-glycoprotein-	9.70E-01	4150000	4050000	4130000	3960000	``	1.00	0.95

Q96PD5	N-acetylmuramoyl-L-alanine amidase	9.70E-01	1540000	1540000	1520000	1480000	1.00	0.99	0.96
P05155	Plasma protease C1 inhibitor	9.70E-01	2130000	2020000	2020000	2100000	0.95	0.95	0.99
P05452	Tetranectin	9.70E-01	789000	780000	788000	815000	0.99	1.00	1.03
Q16610	Extracellular matrix protein 1	9.70E-01	950000	921000	925000	913000	0.97	0.97	0.96
Q96KN2	Beta-Ala-His dipeptidase	9.70E-01	638000	610000	614000	616000	0.96	0.96	0.97
P02747	Complement C1q subcomponent subunit C	9.70E-01	1260000	1200000	1240000	1230000	0.95	0.98	0.98
P67936	Tropomyosin alpha-4 chain	9.70E-01	398000	383000	385000	383000	0.96	0.97	0.96
Q9UBT6	DNA polymerase kappa	9.70E-01	2330000	2320000	2300000	2370000	1.00	0.99	1.02
P32119	Peroxiredoxin-2	9.70E-01	35600	34300	32900	34200	0.96	0.92	0.96
Q13496	Myotubularin	9.70E-01	1190000	1150000	1200000	1150000	0.97	1.01	0.97
Q8WZ75	Roundabout homolog 4	9.70E-01	1050000	1080000	1110000	1030000	1.03	1.06	0.98
Q5VZB9	Doublesex- and mab-3-related transcription factor A1	9.70E-01	71500	68500	67700	67600	0.96	0.95	0.95
Q9BV40	Vesicle-associated membrane protein 8	9.70E-01	511000	464000	474000	456000	0.91	0.93	0.89
P00747	Plasminogen	9.80E-01	14500000	13900000	14200000	14000000	0.96	0.98	0.97
P02774	Vitamin D-binding protein	9.80E-01	23700000	22900000	23800000	23200000	0.97	1.00	0.98
P02675	Fibrinogen beta chain	9.80E-01	3070000	2980000	2980000	2870000	0.97	0.97	0.93
P01023	Alpha-2-macroglobulin	9.80E-01	935000	917000	917000	875000	0.98	0.98	0.94
Q6S8J3	POTE ankyrin domain family member E	9.80E-01	2880000	2800000	2870000	2890000	0.97	1.00	1.00
Q9UGM5	Fetuin-B	9.80E-01	393000	382000	386000	392000	0.97	0.98	1.00
P04070	Vitamin K-dependent protein C	9.80E-01	630000	647000	652000	625000	1.03	1.03	0.99
P49908	Selenoprotein P	9.80E-01	271000	268000	266000	264000	0.99	0.98	0.97
P49747	Cartilage oligomeric matrix protein	9.80E-01	295000	284000	293000	278000	0.96	0.99	0.94
P22105	Tenascin-X	9.80E-01	1770000	1740000	1780000	1750000	0.98	1.01	0.99
P02533	Keratin, type I cytoskeletal 14	9.80E-01	509000	506000	487000	503000	0.99	0.96	0.99
P13646	Keratin, type I cytoskeletal 13	9.80E-01	458000	441000	450000	457000	0.96	0.98	1.00
Q9NPH3	Interleukin-1 receptor accessory protein	9.80E-01	1200000	1170000	1160000	1170000	0.98	0.97	0.98
Q8TF30	WASP homolog-associated protein with actin, membranes and mic	9.80E-01	2070000	2130000	2110000	2060000	1.03	1.02	1.00
Q15113	Procollagen C-endopeptidase enhancer 1	9.80E-01	96800	92300	94100	96800	0.95	0.97	1.00
P33981	Dual specificity protein kinase TTK	9.80E-01	1860000	1870000	1810000	1870000	1.01	0.97	1.01
Q9NZR1	Tropomodulin-2	9.80E-01	1750000	1680000	1730000	1660000	0.96	0.99	0.95
O75717	WD repeat and HMG-box DNA-binding protein 1	9.80E-01	6490000	6200000	6370000	6230000	0.96	0.98	0.96
P18887	DNA repair protein XRCC1	9.80E-01	787000	752000	776000	759000	0.96	0.99	0.96
P24593	Insulin-like growth factor-binding protein 5	9.80E-01	638000	618000	618000	611000	0.97	0.97	0.96
Q8NCN5	Pyruvate dehydrogenase phosphatase regulatory subunit, mitocho	9.80E-01	216000	218000	229000	190000	1.01	1.06	0.88
P30041	Peroxiredoxin-6 spot 12	9.80E-01	6671.64	6623.73	5851.12	6251.84	0.99	0.88	0.94
Q14624	Inter-alpha-trypsin inhibitor heavy chain H4	9.90E-01	11100000	10800000	10900000	10900000	0.97	0.98	0.98
P00751	Complement factor B	9.90E-01	18200000	17400000	17800000	17600000	0.96	0.98	0.97
P02748	Complement component C9	9.90E-01	2680000	2640000	2740000	2660000	0.99	1.02	0.99
P01024	Complement C3	9.90E-01	7180000	7130000	7240000	7070000	0.99	1.01	0.98
P07358	Complement component C8 beta chain	9.90E-01	1840000	1810000	1830000	1760000	0.98	0.99	0.96
P05156	Complement factor I	9.90E-01	2160000	2140000	2180000	2120000	0.99	1.01	0.98
P08697	Alpha-2-antiplasmin	9.90E-01	3470000	3400000	3440000	3450000	0.98	0.99	0.99
Q06033	Inter-alpha-trypsin inhibitor heavy chain H3	9.90E-01	4660000	4600000	4710000	4600000	0.99	1.01	0.99
P35858	Insulin-like growth factor-binding protein complex acid labile subu	9.90E-01	1110000	1080000	1090000	1070000	0.97	0.98	0.96
P20742	Pregnancy zone protein	9.90E-01	2600000	2540000	2520000	2480000	0.98	0.97	0.95
P22792	Carboxypeptidase N subunit 2	9.90E-01	1690000	1700000	1740000	1720000	1.01	1.03	1.02
P13645	Keratin, type I cytoskeletal 10	9.90E-01	1990000	2000000	1920000	2020000	1.01	0.96	1.02
P00488	Coagulation factor XIII A chain	9.90E-01	1340000	1350000	1290000	1310000	1.01	0.96	0.98
Q96IY4	Carboxypeptidase B2	9.90E-01	374000	373000	367000	377000	1.00	0.98	1.01
P43251	Biotinidase	9.90E-01	402000	401000	393000	389000	1.00	0.98	0.97
P02652	Apolipoprotein A-II	9.90E-01	315000	412000	433000	381000	1.31	1.37	1.21
P48740	Mannan-binding lectin serine protease 1	9.90E-01	1050000	1010000	1030000	1010000	0.96	0.98	0.96
P35527	Keratin, type I cytoskeletal 9	9.90E-01	1390000	1380000	1420000	1360000	0.99	1.02	0.98
P27918	Properdin	9.90E-01	238000	239000	245000	243000	1.00	1.03	1.02
P22352	Glutathione peroxidase 3	9.90E-01	174000	177000	176000	177000	1.02	1.01	1.02
P08519	Apolipoprotein(a)	9.90E-01	145000	144000	136000	143000	0.99	0.94	0.99
Q6KC79	Nipped-B-like protein	9.90E-01	3920000	3920000	3920000	3830000	1.00	1.00	0.98
P04275	von Willebrand factor	9.90E-01	2530000	2460000	2430000	2470000	0.97	0.96	0.98
O75912	Diacylglycerol kinase iota	9.90E-01	1050000	1040000	1060000	1050000	0.99	1.01	1.00
Q01546	Keratin, type II cytoskeletal 2 oral	9.90E-01	923000	911000	911000	933000	0.99	0.99	1.01
O14786	Neuropilin-1	9.90E-01	1360000	1300000	1320000	1310000	0.96	0.97	0.96
P05787	Keratin, type II cytoskeletal 8	9.90E-01	2100000	2190000	2120000	2120000	1.04	1.01	1.01
Q15233	Non-POU domain-containing octamer-binding protein	9.90E-01	1380000	1360000	1390000	1390000	0.99	1.01	1.01
Q5H8C1	FRAS1-related extracellular matrix protein 1	9.90E-01	2290000	2330000	2360000	2320000	1.02	1.03	1.01
P58107	Epiplakin	9.90E-01	3380000	3250000	3340000	3350000	0.96	0.99	0.99
Q9BWP8	Collectin-11	9.90E-01	868000	860000	888000	837000	0.99	1.02	0.96
Q7Z2Q7	Leucine-rich repeat-containing protein 70	9.90E-01	676000	677000	668000	650000	1.00	0.99	0.96
Q96I24	Far upstream element-binding protein 3	9.90E-01	1570000	1590000	1520000	1500000	1.01	0.97	0.96
O14917	Protocadherin-17	9.90E-01	2100000	1960000	2000000	1960000	0.93	0.95	0.93
P00450	Ceruloplasmin	1.00E+00	29400000	29400000	29100000	28000000	1.00	0.99	0.95
P06727	Apolipoprotein A-IV	1.00E+00	14100000	13400000	13600000	14000000	0.95	0.96	0.99
P43652	Afamin	1.00E+00	5210000	4830000	5120000	5100000	0.93	0.98	0.98
P10643	Complement component C7	1.00E+00	1400000	1360000	1390000	1380000	0.97	0.99	0.99
P01019	Angiotensinogen	1.00E+00	2180000	2190000	2170000	2070000	1.00	1.00	0.95
P04196	Histidine-rich glycoprotein	1.00E+00	1220000	1180000	1200000	1180000	0.97	0.98	0.97
P05543	Thyroxine-binding globulin	1.00E+00	745000	712000	729000	713000	0.96	0.98	0.96
P51884	Lumican	1.00E+00	2300000	2250000	2280000	2400000	0.98	0.99	1.04
P02750	Leucine-rich alpha-2-glycoprotein	1.00E+00	1060000	1070000	1120000	1060000	1.01	1.06	1.00
P08185	Corticosteroid-binding globulin	1.00E+00	852000	841000	872000	821000	0.99	1.02	0.96
Q14520	Hyaluronan-binding protein 2	1.00E+00	1010000	1000000	1010000	997000	0.99	1.00	0.99
P02741	C-reactive protein	1.00E+00	205000	202000	209000	205000	0.99	1.02	1.00
Q15848	Adiponectin	1.00E+00	65900	68200	64800	64900	1.03	0.98	0.98
P16070	CD44 antigen	1.00E+00	2540000	2510000	2530000	2490000	0.99	1.00	0.98
P06733	Alpha-enolase	1.00E+00	672000	659000	667000	654000	0.98	0.99	0.97
Q14999	Cullin-7	1.00E+00	881000	872000	864000	870000	0.99	0.98	0.99
Q9Y666	Solute carrier family 12 member 7	1.00E+00	1400000	1380000	1360000	1400000	0.99	0.97	1.00
P62158	Calmodulin	1.00E+00	185000	185000	185000	190000	1.00	1.00	1.03
Q86VB7	Scavenger receptor cysteine-rich type 1 protein M130	1.00E+00	2130000	2170000	2200000	2140000	1.02	1.03	1.00
P57789	Potassium channel subfamily K member 10	1.00E+00	793000	735000	726000	742000	0.93	0.92	0.94
O14717	tRNA (cytosine(38)-C(5))-methyltransferase	1.00E+00	729000	757000	729000	736000	1.04	1.00	1.01
O95721	Synaptosomal-associated protein 29	1.00E+00	371000	383000	395000	394000	1.03	1.06	1.06

Figure S1

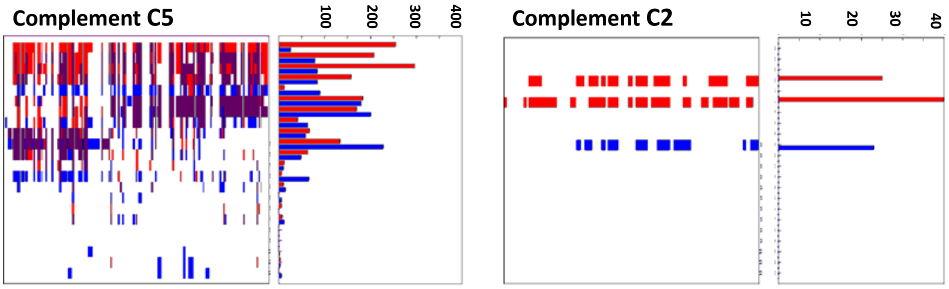

Figure S2

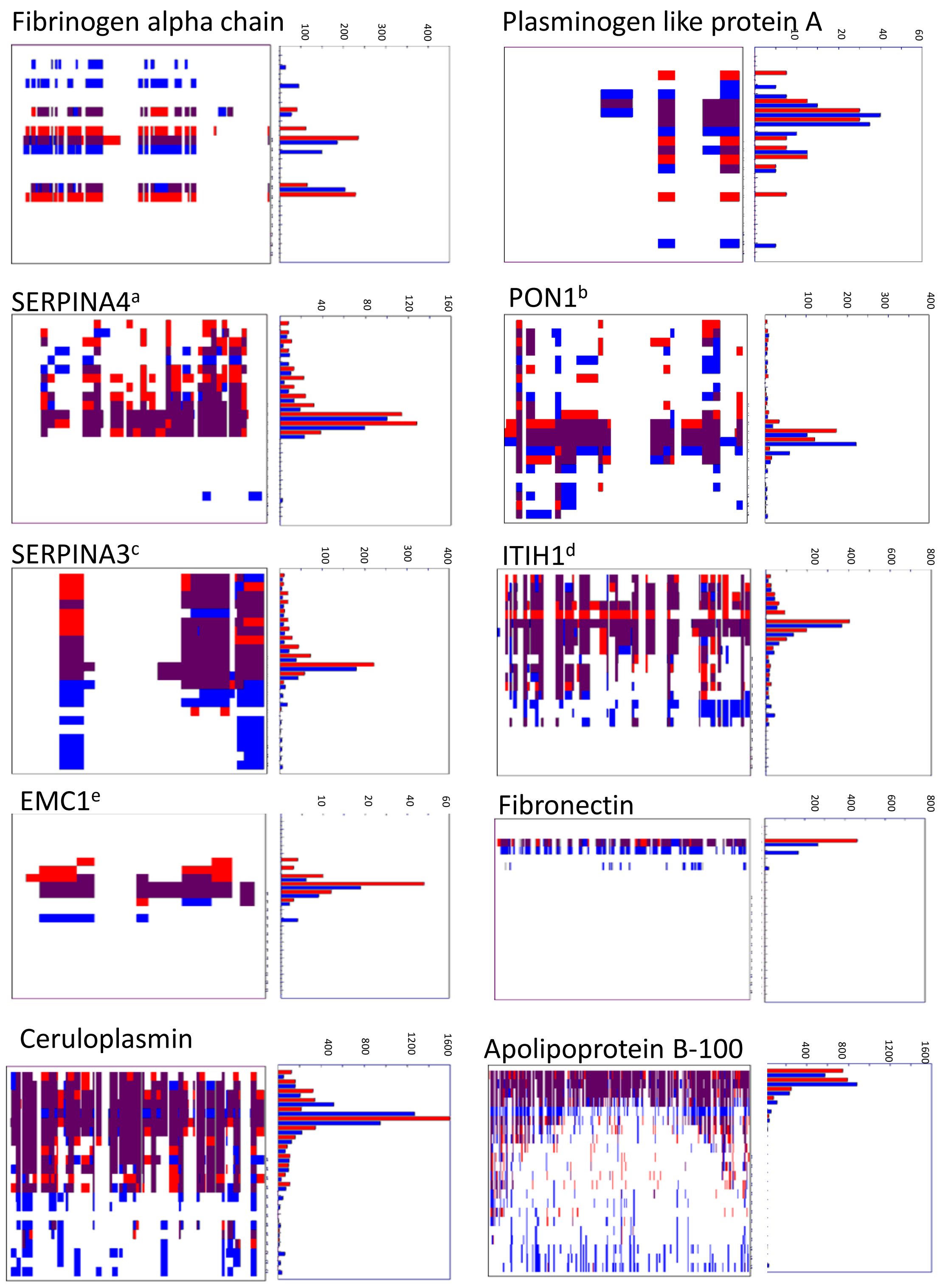

[a]Corticosteroid-binding globulin, [b]Serum paraoxonase, [c]Alpha-1 antichymotrypsin, [d]Inter alpha trypsin inhibitor, [e] Extracellular matrix protein-1
